# Classification of weevils as a data-driven science: leaving opinion behind

**DOI:** 10.3897/zookeys.439.8391

**Published:** 2014-09-08

**Authors:** Bjarte H. Jordal, Sarah M. Smith, Anthony I. Cognato

**Affiliations:** 1 University of Bergen, University Museum of Bergen, PB 7800, NO-5020 Bergen, Norway; 2 Michigan State University, Department of Entomology, 288 Farm Lane, East Lansing, MI 48824, United States of America

**Keywords:** Curculionoidea, Scolytinae, Platypodinae, weevil phylogeny, taxonomic naming criteria, Evolutionary systematics, Scolytidae, Platypodidae

## Abstract

Data and explicit taxonomic ranking criteria, which minimize taxonomic change, provide a scientific approach to modern taxonomy and classification. However, traditional practices of opinion-based taxonomy (i.e., mid-20^th^ century evolutionary systematics), which lack explicit ranking and naming criteria, are still in practice despite phylogenetic evidence. This paper discusses a recent proposed reclassification of weevils that elevates bark and ambrosia beetles (Scolytinae and Platypodinae) to the ranks of Family. We demonstrate that the proposed reclassification 1) is not supported by an evolutionary systematic justification because the apparently unique morphology of bark and ambrosia beetles is shared with other unrelated wood-boring weevil taxa; 2) introduces obvious paraphyly in weevil classification and hence violates good practices on maintaining an economy of taxonomic change; 3) is not supported by other taxonomic naming criteria, such as time banding. We recommend the abandonment of traditional practices of an opinion-based taxonomy, especially in light of available data and resulting phylogenies.

## Introduction

Catalogues of plant and animal species are for many scientists essential tools in biodiversity related research, ecology and wildlife management. Publications of this nature include the compilation of large amounts of data from thousands of different literature sources. Without the time and effort devoted to such research activity, most evolutionary and ecological studies are undoubtedly more difficult given the fragmented distribution of literature relevant to any projects on a particular group of organisms. Major taxonomic reviews and taxonomic catalogues organize their contents according to a classification scheme chosen by the author, which may not follow the best evidence for higher level relationships. This creates an unfortunate situation as comprehensive catalogues are frequently cited sources for taxon relationships and as such, may misrepresent the evolution of a group of organisms.

In a recent supplement to the catalogue on the worldwide fauna and taxonomy of Scolytinae and Platypodinae (bark and ambrosia beetles), Bright (2014) delivers a much needed resource on these groups of weevils. This third supplemental volume to the main catalogue (Wood and Bright 1992) contains references to recently published information on a large number of species and higher taxa. As in previous volumes by the same author (Bright and Skidmore 1997, 2002), the level of detail and accuracy is impressive, and presents a very important contribution towards efficient biodiversity and taxonomic research. Within this publication, Bright also presents a radically new classification based on evolutionary systematic philosophy of the mid-20^th^ century (Mayr et al. 1953), including groups of tribes elevated to new subfamilies which are at odds with the current phylogenetic knowledge of these beetles, and reintroduces the archaic scheme that gives Scolytinae and Platypodinae family ranks outside Curculionidae.

Our philosophical debate began over 50 years ago with the growing use of phylogenies to infer classifications. The greatest arguments occurred between the evolutionary systematists who recognized taxa and their rank based on evolutionary uniqueness, including paraphyletic groups, and the cladists (phylogeneticists) who recognized monophyletic (i.e., holophyletic) taxa and their rank based on group hierarchy (Wiley 1979). Currently, there is a consensus among systematists that monophyly is the most important criterion for the recognition of taxa because the resulting taxonomic classification has evolutionary context (Wiley and Lieberman 2011). Unfortunately, most taxonomists have not been explicit about their criteria for naming taxa at various ranks and have been content to leave the decision to their expert opinion. However, explicit taxonomic naming conventions or criteria would help remove this subjectivity (Vences et al. 2013; Wiley and Lieberman 2011). Three primary criteria assure that named groups are monophyletic and well-supported, phenotypically identifiable, and promote an economy of nomenclatural change (Vences et al. 2013). In addition, several secondary criteria, such as time banding, have been suggested as helpful in the recognition of ranks (Vences et al. 2013). As we review here, there is ample data that support the monophyly of scolytines and platypodines and these groups are phenotypically identifiable. The issue is the recognition of these groups as families because this solution does not promote an economy of nomenclatural change when the ranks of other weevil groups are considered.

We argue that the application of family category on these two weevil groups is unjustified because: i) evolutionary systematic justification for family rank is unsupported, i.e., the apparently unique morphology of bark and ambrosia beetles is in part shared with other unrelated wood-boring weevil taxa, ii) the suggested classification does not promote an economy of nomenclatural change, i.e., it creates massive paraphyly of the remaining Curculionidae; and, iii) the suggested classification is not supported by other taxonomic naming criteria, i.e., it elevates two relatively young lineages of weevils to the same rank as much older groups.

**Table 1.** List of taxa mentioned in the text, with author and year of publication.

Name	Author & date
Anthonomini	Thompson 1859
Araucariini, *Araucarius*	Kuschel 1966
Attelabidae	Billberg 1820
Bagoinae	Thompson 1859
Baridinae	Schoenherr 1836
Bostrichidae	Erichson 1836
Brachyceridae, -inae	Billberg 1820
Brentidae	Billberg 1820
Conoderinae	Schoenherr 1833
Cossoninae	Schoenherr 1825
Cryphalinae	Lindemann 1877
*Cryphalus*	Erichson 1836
Curculionoidea, -idea, -inae	Latreille 1802
Cyclominae	Schoenherr 1826
*Dactylipalpus*	Chapuis 1869
Dryocoetini	Lindemann 1877
Dryophthoridae, -inae	Schoenherr 1825
Entiminae	Schoenherr 1823
Hexacolidae, -inae, ini	Eichhoff 1878
*Homoeometamelus*	Hustache 1936
*Hylastes*	Erichson 1836
Hylesininae	Erichson 1836
*Hylurgops*	LeConte 1876
Hyorrhynchini	Hopkins 1915
Hyperinae	Marseul 1863
*Hypocryphalus*	Hopkins 1915
Ipinae, -ini	Bedel 1888
Mesoptiliinae	Lacordaire 1863
Molytinae	Schoenherr 1823
*Phrixosoma*	Blandford 1897
Platypodidae, -inae	Shuckard 1840
Premnobiini, -ina	Browne 1962
Scolitarii, Scolytoidea, -idae, -inae, ini,	Latreille 1804
Scolytoplatypodini	Blandford 1893
*Scolytus*	Geoffroy 1762
Xyleborini	LeConte 1876
Xyloctonini	Eichhoff 1878
Xyloterini	LeConte 1876

## History of weevil classification in reference to scolytines and platypodines

Bark and ambrosia beetles were treated separately from other weevils from the beginning of binominal nomenclature (see e.g. Wood (1978) and Alonso-Zarazaga and Lyal (2009) for extensive reviews). Initially, scolytines and platypodines were placed within the family Bostrichini (Erichson 1836; Latreille 1802) and were each later recognized as the families Scolitarii (Latreille 1804) and Platypodidae (Shuckard 1840). Some authors (Eichhoff 1864; Ratzeburg 1837) proposed a non-Linnean nomenclature (Xylophaga), but it was rarely used. After the first major taxonomic review of these beetles (Lacordaire 1865), scolytines and platypodines were viewed either as separate families (Bright 2014; Chamberlin 1939; Chapuis 1869; Schedl 1952; Schedl 1959; Schedl 1972; Wood 1973, 1978), as three families (Lindemann 1877), a superfamily Scolytoidea (Hopkins 1915) that was later adopted by Chamberlin (1939, 1958) and Schedl (e.g. 1941), or as a single family comprised of both scolytines and platypodines (Blandford 1897). Various authors suggested a close relationship between scolytines, platypodines and cossonines and that these taxa were more distantly related to the ‘true weevils’ (Lindemann 1877; Wood 1973), although the view of scolytines as weevils was previously proposed (Latreille 1806).

Crowson (1955) proposed a radically different relationship by placing each of the Platypodinae and Scolytinae as subfamilies of Curculionidae – the ‘advanced weevils’ which possess geniculate antennae. The new scheme was adopted by other leading Coleopterists such as Lawrence and Newton (1995), and weevil specialists, e.g. Thompson (1992), Zimmerman (1993), Kuschel (1995), and Oberprieler et al. (2007). Alonso-Zarazaga and Lyal (1999) viewed scolytines as a subfamily of Curculionidae but recognized platypodines as a family, an opinion that they later changed (2009) following Oberprieler et al. (2007). This classification was supported by a wide range of morphological characters, particularly from the larvae (Lekander 1968; May 1993; Viedma 1963), and was supported by phylogenetic analyses of both adult and larval character (Kuschel 1995; Marvaldi 1997). The original Crowson scheme therefore has been adopted with only minor emendations in worldwide databases such as ITIS, GBiF, NCBI and EoL. Current disagreement is mainly confined to the number of subfamilies in Curculionidae, and the status of Brachycerinae (-idae) and Dryophthorinae (-idae) (Table 2).

**Table 2.** Comparison of weevil classification of extant families as more broadly defined by Oberprieler et al. (2007) and more narrowly defined by Alonso-Zarazaga and Lyal (1999).

Oberprieler et al. 2007		Alonso-Zarazaga and Lyal 1999	
Nemonychidae	Nemonychinae	Nemonychidae	Nemonychinae
	Cimberidinae		Cimberidinae
	Rhinorhynchinae		Rhinorhynchinae
Anthribidae	Anthribinae	Anthribidae	Anthribinae
	Choraginae		Choraginae
	Urodontinae		Urodontinae
Belidae	Belinae	Belidae	Belinae
	Oxycoryninae		Oxycoryninae
Attelabidae	Attelabinae	Attelabidae	Attelabinae
	Rhynchitinae		Rhynchitinae
			Archolabinae
			Isotheinae
			Pterocolinae
		Eurhynchidae	Eurhynchinae
Caridae	Carinae	Caridae	Carinae
Brentidae	Brentinae	Brentidae	Brentinae
	Apioninae		Antliarhininae
	Eurhynchinae		Cyladinae
	Ithycerinae		Cyphagoginae
	Microcerinae		Pholidochlamydinae
	Nanophyinae		Taphroderinae
			Trachelizinae
			Ulocerinae
		Nanophyidae	Nanophyinae
		Ithyceridae	Ithycerinae
		Apionidae	Apioninae
			Myrmacicelinae
			Rhinorhynchidiinae
**Curculionidae**	Brachycerinae	Brachyceridae	Brachycerinae
			Microcerinae
			Ocladiinae
		Erirhinidae	Erirhininae
			Tadiinae
		Raymondionymidae	Raymondionymidae
			Myrtonyminae
		Cryptolaryngidae	Cryptolarynginae
	Dryophthorinae	Dryophthoridae	Dryophthorinae
			Cryptodermatinae
			Orthognathinae
			Stromboscerinae
			Rhynchophorinae
	Entiminae	**Curculionidae**	Entiminae
	Curculioninae		Curculioninae
	Baridinae		Baridinae
			Conoderinae
			Ceutorhynchinae
	Molytinae		Molytinae
			Cryptorhynchinae
			Magdalinae
			Mesoptiliinae
			Lixinae
	Cyclominae		Cyclominae
			Hyperinae
			Bagoinae
	Cossoninae		Cossoninae
	Scolytinae		Scolytinae
			(2009: Platypodinae)
	Platypodinae	Platypodidae	

While entomologists in general have accepted the modern definition of Curculionidae, many forest entomologists that actively work on bark and ambrosia beetle ecology and forest health tend to oppose Crowson’s system. The most prominent opponent was Stephen L. Wood who published a series of influential monographs and reviews (Wood 1973, 1978, 1982, 1986, 1993, 2007; Wood and Bright 1992). Wood argued for a close relationship between Scolytinae and Platypodinae and placed them outside Curculionidae, closer to the origin of the more primitive weevil lineages. However, much of his evidence came from a rather biased selection of characters, mainly from the head region and Wood’s (2007) desire to recognize their striking phenotypic differences (see also Morimoto and Kojima 2003). A number of concurrent publications rejected Wood’s hypothesis, and clearly showed that scolytines and platypodines were nested within Curculionidae, hence the subfamily rank.

## Weevil phylogenetics

**Figure 1.**
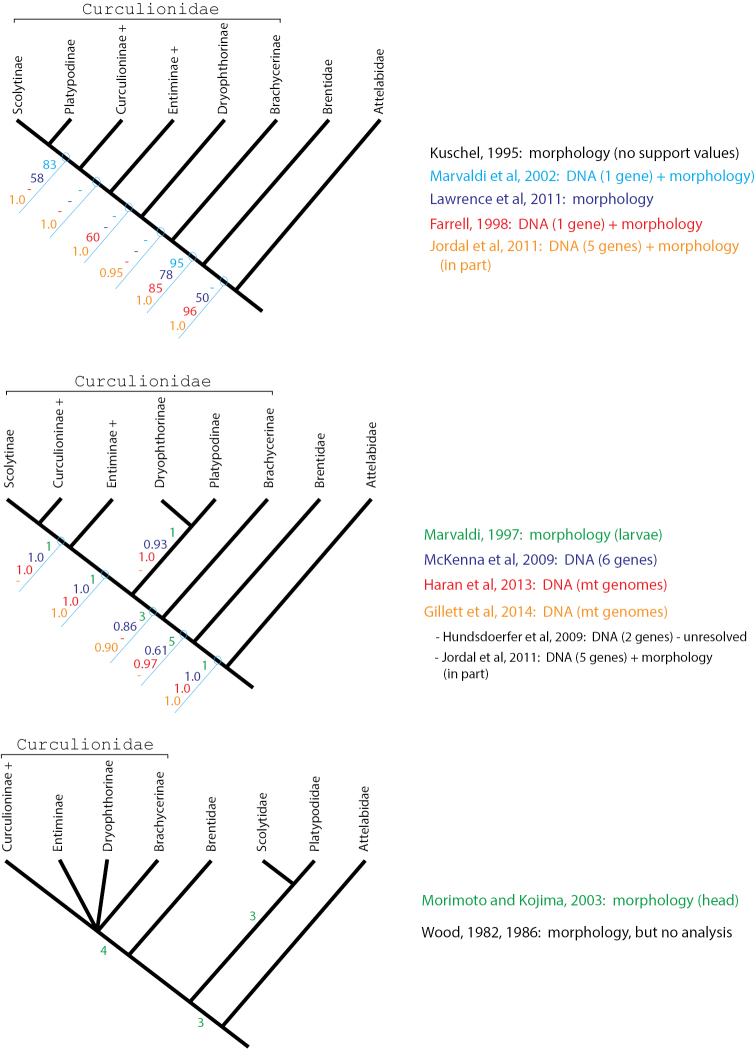
Three alternative phylogeny-based classifications. Numbers on nodes indicate support values according to the method reported in the publication listed in the same colour to the right. Low integers (1-9) indicate Bremer support or number of apomorphic characters, higher integers (>50) indicate parsimony bootstrap support, and proportions (>0.50) indicate posterior probabilities from Bayesian analyses.

This brings us to the crux of the matter, namely that weevil relationships and rank can only be objectively assessed through the inclusion of the broadest possible range of characters in a phylogenetic analysis. Bright’s change in rank for bark and ambrosia beetles is not based on carefully designed hypothesis testing of monophyly, but through the use of arguments, similar to Wood (1986), which cite certain sets of possibly uniquely derived morphological characters to justify the rank of family (Wood 1973, 1978, 1982, 1986, 1993, 2007). This evolutionary systematic perspective does not fully consider the results of weevil phylogenetic studies, which are based on large and fairly unbiased data sets. The resulting phylogenies from these inclusive datasets demonstrate the monophyly of Scolytinae and Platypodinae and their placement within Curculionidae (Fig. 1). The nested position in Curculionidae is supported by morphology-based (Kuschel 1995; Lawrence et al. 2011; Marvaldi 1997) as well as molecular-based phylogenetic studies (Gillett et al. 2014; Haran et al. 2013; Hundsdoerfer et al. 2009; McKenna et al. 2009), and combined morphological and molecular studies which included thousands of nucleotides from 5-6 genes (nuclear and mitochondrial) and hundreds of morphological characters (Farrell 1998; Jordal et al. 2011; Marvaldi et al. 2002). The placement of some Curculionidae subfamilies is still uncertain due to their relatively simultaneous origin (see Gillett et al. 2014; Jordal et al. 2011; McKenna et al. 2009), but all studies clearly indicate a nested position of Scolytinae within a narrowly defined Curculionidae (*sensu* Alonso-Zarazaga and Lyal 1999).

**Figure 2.**
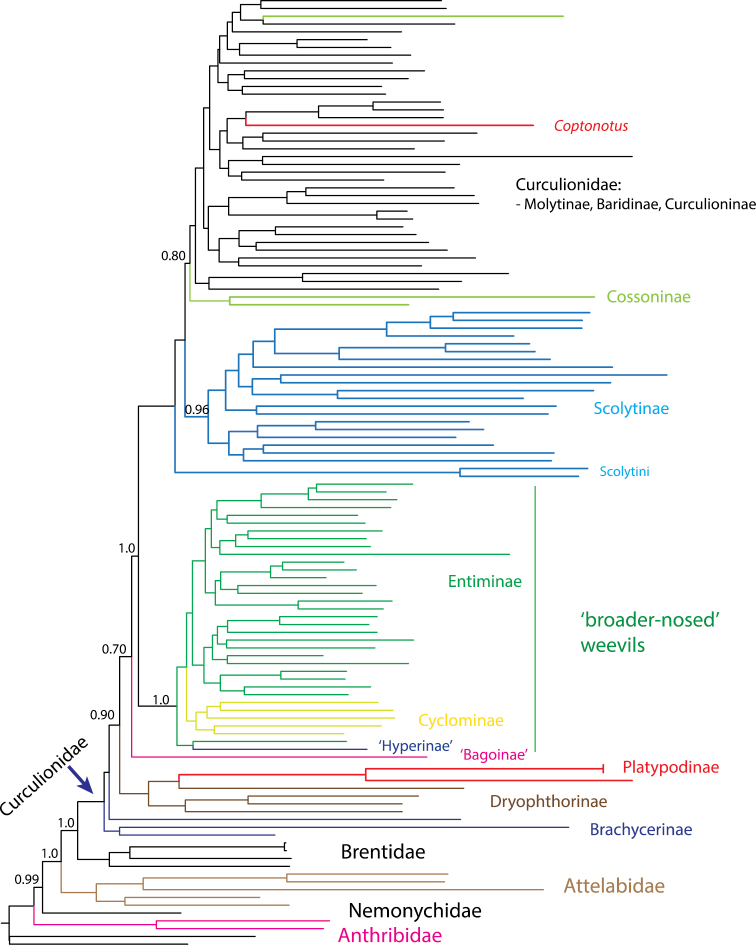
Mitochondrial genome phylogeny redrawn from Gillett et al. (2014), with various families and subfamilies marked in different colours. Node support values are posterior probabilities >0.70.

Platypodinae may also belong to a similarly defined Curculionidae, but the long phylogenetic branches that characterise Platypodinae make placement of this subfamily less certain. In several purely molecular phylogenetic studies, they tend to group with Dryophthorinae, but still well inside a more broadly defined Curculionidae (*sensu* Oberprieler et al. 2007) that includes Brachycerinae and Dryophthorinae (Fig. 2). The family status of Platypodinae has been suggested (e.g. Thompson 1992) and is an issue that potentially interferes less with an economical approach to taxon name changes although the assessment of platypodines is premature given the absence of robust phylogenetic data. Our concerns are therefore mainly with the status of Scolytinae.

## An evolutionary systematic argument for Scolytidae and Platypodidae is unsupported

Bright rejects the current classification scheme for weevils mainly based on what he describes as overwhelming morphological differences between Scolytinae and Platypodinae and the remaining Curculionidae. However, phylogenetic analyses of morphological data do not support his view, and both larval (Marvaldi 1997) and adult characters (Kuschel 1995; Lawrence et al. 2011) support a nested position of Scolytinae and Platypodinae within Curculionidae. Most of the evidence cited by Bright includes head features such as the lack of a rostrum and hypostomal spine, and the pregular sutures defining the pregular sclerite (Wood 1973, 1978). Certainly, if a phylogenetic analysis is based on head features only, and coded according to Wood’s (1973, 1978) interpretation of these features, it will likely result in a more basal position of bark and ambrosia beetles (Morimoto and Kojima 2003). However, Lyal (1995) – in a very detailed anatomical study of the weevil head – clearly refuted this as evidence, showing that pregular sutures are not at all unique and not much different from other advanced weevils with less developed rostrum. He also showed that head features in Platypodinae and Scolytinae are not homologous.

Moreover, Bright argues that socketed denticles on the tibiae are synapomorphic for Scolytinae, which in fact they are not. Socketed denticles are found throughout the insect world in burrowing species, particularly so in wood-boring beetles. Strong socketed denticles along the lateral margins of all tibiae are found in unrelated wood-boring groups such as the conoderine genus *Homoeometamelus* (see Jordal et al. 2011) and in the cossonine genus *Araucarius* (see Mecke 2005). At the other end of this character continuum there are entire scolytine lineages without socketed denticles, such as the Scolytini and most Hyorrhynchini, and in the entire Platypodinae. Furthermore it is incorrect that all scolytines lack corbels on the apical end of the metatibiae. There is clearly an inner flange present between the inner tibial insertion area and the outer edge that is fringed by rough setae or denticles, matching the definition for the open type corbels in *Phrixosoma*, *Dactylipalpus* and *Hylastes*/*Hylurgops* (Jordal 2012).

Bright also referred to differences in larval head features between Scolytinae and other Curculionidae. This is entirely at odds with published sources showing that Scolytinae is indistinguishable from many other Curculionidae based on larval characters (Gardner 1934; Lekander 1968; May 1993; Viedma 1963). The features referred to by Bright are atypical and likely confined to the genus *Scolytus* which actually shows several similarities with larvae in the molytine tribe Mesoptiliini (Lekander 1968; May 1993; Viedma 1963). Phylogenetic analyses including diverse weevil larval characters strongly supports a close relationship between Scolytinae and Cossoninae and to the broad nosed Entiminae, while these three groups are more distantly related to Dryophthorinae and Platypodinae, and even more so to Brentidae (Marvaldi 1997).

Overall, the morphological uniqueness in Scolytinae and Platypodinae fades rapidly when all body parts and all life stages are studied simultaneously in a phylogenetic analysis. The strong arguments for a separate position of Scolytinae and Platypodinae hinges upon the study of few characters which are apparently under strong adaptive selection for optimizing tunnelling behaviour in dead wood. The characters most frequently used to argue for an early separate standing of these groups all appear to be losses or modifications of plesiomorphic features. Optimisation of these features on the best supported phylogenetic topologies (e.g. Fig. 2), demonstrates that the hypostomal teeth are lost multiple times, including certain Cossoninae and Entiminae (Kuschel et al. 2000), the metatibial corbel is lost in connection with a strongly flattened tibiae as in Cossoninae and Conoderinae (e.g. Thompson 1992), particularly in the Araucariini and the wood boring conoderine genus *Homoeometamelus* (Jordal et al. 2011; Mecke 2005), and the rostrum is strongly reduced to entirely absent in many wood boring cossonines (Jordal 2014).

## The recognition of Scolytidae and Platypodidae does not support an economy of taxonomic change

The recognition of Scolytidae, and in most classification schemes also Platypodidae, would render Curculionidae paraphyletic and as a result create more nomenclatural issues and work for current and subsequent weevil taxonomists. In order to maintain monophyly of Curculionidae, many if not most current weevil subfamilies would need to change rank to family given the phylogenetic position of scolytines and platypodines (Fig. 2). Some of these subfamilies are paraphyletic; thus, their change in rank would require the recognition of additional currently unnamed clades as families. As illustrated by the most recent and well sampled study to date (Fig. 2), the mitochondrial genome phylogeny indicates a separate clade of the ‘broader-nosed’ weevils (Entiminae, Cyclominae, Hyperinae) as sister to *Scolytus* (Scolytini), the remaining Scolytinae, and most other Curculionidae except Brachycerinae, Dryophthorinae and Platypodinae. This means that the erection of Scolytinae to a family would require a similar elevation in status for several Curculionidae subfamilies as families (e.g. Entiminae, Cyclominae and Hyperinae) to restore the monophyletic status of Curculionidae. Without a coordinated change in ranks of equivalent weevil groups, the isolated act on Scolytinae and Platypodinae will cause instability in weevil classification.

There is still much phylogenetic ambiguity in even the most well-sampled weevil phylogenies, thus with greater phylogenetic resolution in future analyses, many of these new recognized families would likely be demoted in rank or synonymized and forgotten. The recognition of Scolytidae and Platypodidae also results in the loss of taxonomic information. As families these groups can only be inferred as beetles with some distinguishing characters. But as weevil subfamilies, these groups are recognised as distinguished weevils, namely as snout-less.

In addition, with the elevation of Scolytinae to full family status, Bright promotes 13 new subfamilies, 10 containing a single tribe, and 3 with a collection of 2, 6 or 12 tribes. Even if everyone accepted ‘Scolytidae’, the change in categories is premature. Bright states that “the ultimate goal of phylogenetic systematics is the development and recognition of monophyletic lineages. As stated above, I herein recognize 13, supposedly monophyletic, subfamilies.” However, he does not cite a phylogeny or discuss synapomorphic characters that would support his supposition of monophyletic subfamilies. Although we share Bright’s view that Wood’s (1986) system includes many paraphyletic and polyphyletic groups, we do not see the evidence presented for how Bright’s alternative groupings should increase the number of monophyletic taxa. Published Scolytinae phylogenies generally lack the phylogenetic resolution to suggest a stable classification based on monophyly. Jordal and Cognato’s phylogeny (2012) is the best sampled phylogeny to date (200 taxa; 4,000 bp from 5 genes) and still many intergeneric and intertribal relationships are unresolved. There is no evidence for the monophyly of Bright’s proposed subfamilies Hexacolinae (phylogenetic data indicate paraphyly with respect to Scolytoplatypodini), Hylesininae (a mixture of unrelated tribes and genera), Ipinae (Xyloctonini and Xyloterini belong elsewhere), and Cryphalinae (*Cryphalus* and *Hypocryphalus* distinctly different from other Cryphalini).

There are also issues concerning monophyly and their corresponding category. Bright does not include criteria for deciding which monophyletic groups should be considered subfamilies. We assume his decision is based on large differences among morphological features (a main tenant in evolutionary systematic philosophy) but the classification is subjective without quantifying these differences. For example, Cactopinini and Micracidini are sister (or nested) clades (Jordal and Cognato 2012; Jordal et al. 2008). Bright proposed separate subfamilies for these groups, but one could justify placing both tribes in one subfamily. Similarly, nomenclatural revision that combines the ranks of Xyleborini and Dryocoetini appears necessary. This is the group where most detailed research has been done, showing that both morphological and molecular data strongly support a nested position of xyleborines within the dryocoetine clade (Farrell et al. 2001; Jordal et al. 2002; Jordal and Cognato 2012; Normark et al. 1999). The same applies to Premnobiini which was recently moved to Ipini as Premnobiina based on molecular and morphological evidence in a phylogenetic context (Cognato 2013).

## Other taxonomic naming criteria do not support the recognition of Scolytidae and Platypodidae

Of the other proposed taxonomic naming criteria, time banding (the use of evolutionary age to determine rank) is most applicable to this issue (Vences et al. 2013). Bright suggests that the origin of scolytines occurred in the late Jurassic and derived from “basal” Curculionoidea families such as Brentidae or Attelabidae. Neither the hierarchical structure (Fig. 2) nor molecular dating of weevils suggests that Scolytinae and Platypodinae are derived from these groups or from other groups of comparable age (Farrell 1998; Jordal et al. 2011; McKenna et al. 2009). While these more primitive weevil clades originated in the early Cretaceous or late Jurassic, Scolytinae and Platypodinae are more derived in the molecular analyses and hence much younger lineages of mid-Cretaceous origin.

The oldest scolytine and platypodine fossils are both of mid-Cretaceous age around 100 (Burmese amber) and 116 Ma (Lebanese amber), and fit nicely with these time estimates (Cognato and Grimaldi 2009; Kirejtshuk et al. 2009). Although the weevil fossil record is not particularly rich, it nevertheless follows a sequence of older basal non-geniculate weevils in early Cretaceous deposits, with more modern geniculate forms appearing no earlier than in the mid-Cretaceous. The fossil records in Scolytinae or in Platypodinae are not older than other Curculionidae, including Curculioninae. A fossil of the latter subfamily was recently discovered from the Santana formation in Brazil, likely a member of the tribe Anthonomini, which again indicates a minimum age of 116 Ma for this fairly modern group of weevils (Santos et al. 2011). These fossil ages seems to be close to the maximum age for the advanced weevils as indicated by the shallow phylogenetic internodes characterising the entire clade consisting of Scolytinae, Molytinae, Cossoninae, Baridinae and Curculioninae and related subfamilies or tribes, which implies a rapid radiation just after the origin of the broad nosed weevils (Entiminae, Cyclominae, Hyperinae) (Gillett et al. 2014; Jordal et al. 2011; McKenna et al. 2009).

## Recommendations

For the 21^st^ century, taxonomic classification should be based on well-supported, character-rich phylogenies and clear taxonomic ranking (naming) criteria. Instead, the newly proposed classification scheme is derived from an evolutionary systematic perspective, which, despite the phylogenetic evidence to the contrary, is biased by a selection of apparently unique characters. The resulting high cost of change to Curculionidae taxonomy further undermines the proposed classification. We strongly recommend current and subsequent researchers to evaluate classifications conservatively to maintain stability and encourage an economy of taxonomic change that is based on well-supported phylogenies reconstructed with various sources of data. Awaiting the great overhaul of curculionid classifications, the catalogue published by Alonso-Zarazaga and Lyal (1999, 2009) best preserves nomenclatural stability by heeding to the current phylogenetic evidence and by maintaining a link to well-established Scolytinae tribes *sensu* Wood (1978, 1986). We understand that many users of weevil classification are comfortable with the tradition of subjective assessment and authority in taxonomy. We, on the other hand, do not see comfort in tradition, and would like to see modern scolytine taxonomy evolve into a data-driven science guided by explicit taxonomic naming criteria.
